# Multiscale Mechanical Model of the Pacinian Corpuscle Shows Depth and Anisotropy Contribute to the Receptor’s Characteristic Response to Indentation

**DOI:** 10.1371/journal.pcbi.1004370

**Published:** 2015-09-21

**Authors:** Julia C. Quindlen, Victor K. Lai, Victor H. Barocas

**Affiliations:** 1 Department of Biomedical Engineering, University of Minnesota, Minneapolis, Minnesota, United States of America; 2 Department of Chemical Engineering, University of Minnesota, Duluth, Minnesota, United States of America; University of California San Diego, UNITED STATES

## Abstract

Cutaneous mechanoreceptors transduce different tactile stimuli into neural signals that produce distinct sensations of touch. The Pacinian corpuscle (PC), a cutaneous mechanoreceptor located deep within the dermis of the skin, detects high frequency vibrations that occur within its large receptive field. The PC is comprised of lamellae that surround the nerve fiber at its core. We hypothesized that a layered, anisotropic structure, embedded deep within the skin, would produce the nonlinear strain transmission and low spatial sensitivity characteristic of the PC. A multiscale finite-element model was used to model the equilibrium response of the PC to indentation. The first simulation considered an isolated PC with fiber networks aligned with the PC’s surface. The PC was subjected to a 10 μm indentation by a 250 μm diameter indenter. The multiscale model captured the nonlinear strain transmission through the PC, predicting decreased compressive strain with proximity to the receptor’s core, as seen experimentally by others. The second set of simulations considered a single PC embedded epidermally (shallow) or dermally (deep) to model the PC’s location within the skin. The embedded models were subjected to 10 μm indentations at a series of locations on the surface of the skin. Strain along the long axis of the PC was calculated after indentation to simulate stretch along the nerve fiber at the center of the PC. Receptive fields for the epidermis and dermis models were constructed by mapping the long-axis strain after indentation at each point on the surface of the skin mesh. The dermis model resulted in a larger receptive field, as the calculated strain showed less indenter location dependence than in the epidermis model.

## Author Summary

We performed computer simulations of the mechanical behavior of the Pacinian corpuscle (PC), a sensory receptor in the skin that helps detect short-term contact and high-frequency vibration. The PC is composed of a series of tissue layers, and we found that this characteristic structure may explain the response of the PC in indentation experiments. We also found that the deep placement of the PC within the skin allows it to detect stimuli over a wide area of the skin but not to distinguish the specific location of the stimulus. These findings are a step but still an early step towards the broad goal of understanding how mechanical stimuli to the skin are translated into neural signals to the spinal cord and brain, and many open questions still remain about how the different sensors of the nervous system work together to create our sense of touch.

## Introduction

Mechanoreceptors, a major component of the somatosensory system, detect specific physical stimuli and produce neural signals that give rise to sensations such as touch and pain [1]. Cutaneous mechanoreceptors respond to mechanical stimuli and consist of afferent nerve fibers surrounded by specialized end organs that collectively encode a wide range of different touch sensations [2,3]. The Pacinian corpuscle (PC) is a cutaneous mechanoreceptor that responds primarily to vibratory stimuli in the frequency range of 20–1000 Hz [4,5]. The PC has low spatial sensitivity across the surface of the skin, and the receptive field of a single PC may span an entire hand [2]. The PC is located in the dermis of glabrous skin [6,7].

**Fig 1:**
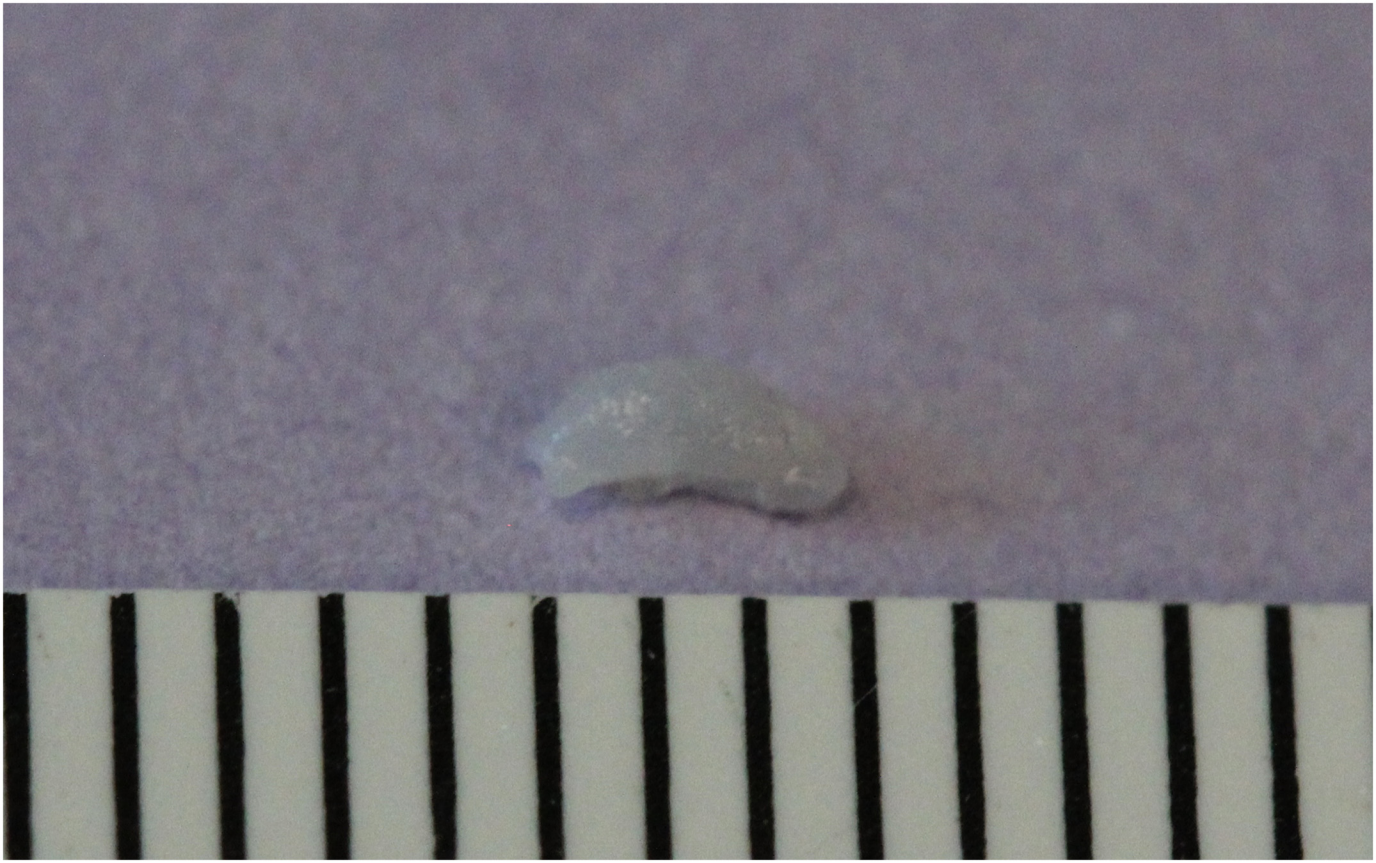
Excised Pacinian corpuscle. Pacinian corpuscle removed from a human cadaveric hand. Tick marks on the ruler correspond to 0.5 mm. Sample was obtained from cadaveric tissue provided, with approval, by the University of Minnesota Anatomy Bequest Program.

The PC has been widely studied because of its relatively large size (Fig 1). The PC is about 1 mm in length by 0.67 mm in width and has an ovoid shape with a single myelinated nerve fiber located along the long axis of the receptor [8,9]. The nerve fiber is surrounded by three main zones: an inner core, which contains bilaterally arranged cytoplasmic lamellae; an intermediate growth zone; and an outer core, which consists of 30 or more concentrically aligned collagenous lamellae [9]. The lamellae are believed to act collectively as a high-pass filter that shields the nerve fiber at the receptor center from low frequency, high amplitude stimuli [2,10,11].

The PC is a difficult organ to understand because its function involves a complex, interrelated set of biological, chemical, mechanical, and electrical phenomena. Much recent work has focused on the development of the PC and identification of relevant transcription factors, such as c-Maf and ER-81 [12,13]. Release of GABA from the inner core has also been suggested to play a role in the rapid adaptivity of the PC [14]. In the electrical and neural engineering arena, Lesniak and Gerling [15] have recently put forward computational models of the tactile mechanosensory system. None of these studies, however, address the fundamental mechanics of the PC and the role of its structure in determining how skin displacement is transmitted to the PC neurite.

The work of Güçlü *et al*.[7] was an important exploration of PC mechanics. They used finite-element modeling to investigate the role of the PC’s geometry in its mechanical response to static indentation. Experimental data in which PCs were indented by cylindrical contactors with step waveforms of various amplitudes were compared to the computational models. Semi-infinite plane and ovoid models produced similar displacements within the PC in response to static indentation, and neither model matched the localization of strain near the contractor seen in the experiment.

The purpose of this study was to test two specific hypotheses about the biomechanics of the PC. First, Güçlü *et al*. rejected the hypothesis that receptor *shape* leads to the observed mechanical behavior of the PC, leaving open the question of how the strain concentrates near the indentation site; herein, we tested the hypothesis that *mechanical anisotropy* contributes to the strain localization. Second, having concluded that a structural model of the PC is mechanically acceptable, we used that model to test the hypothesis that *deep embedding* within the skin contributes to the low spatial sensitivity and large receptive field of the PC.

## Methods

### Multiscale Model

**Fig 2:**
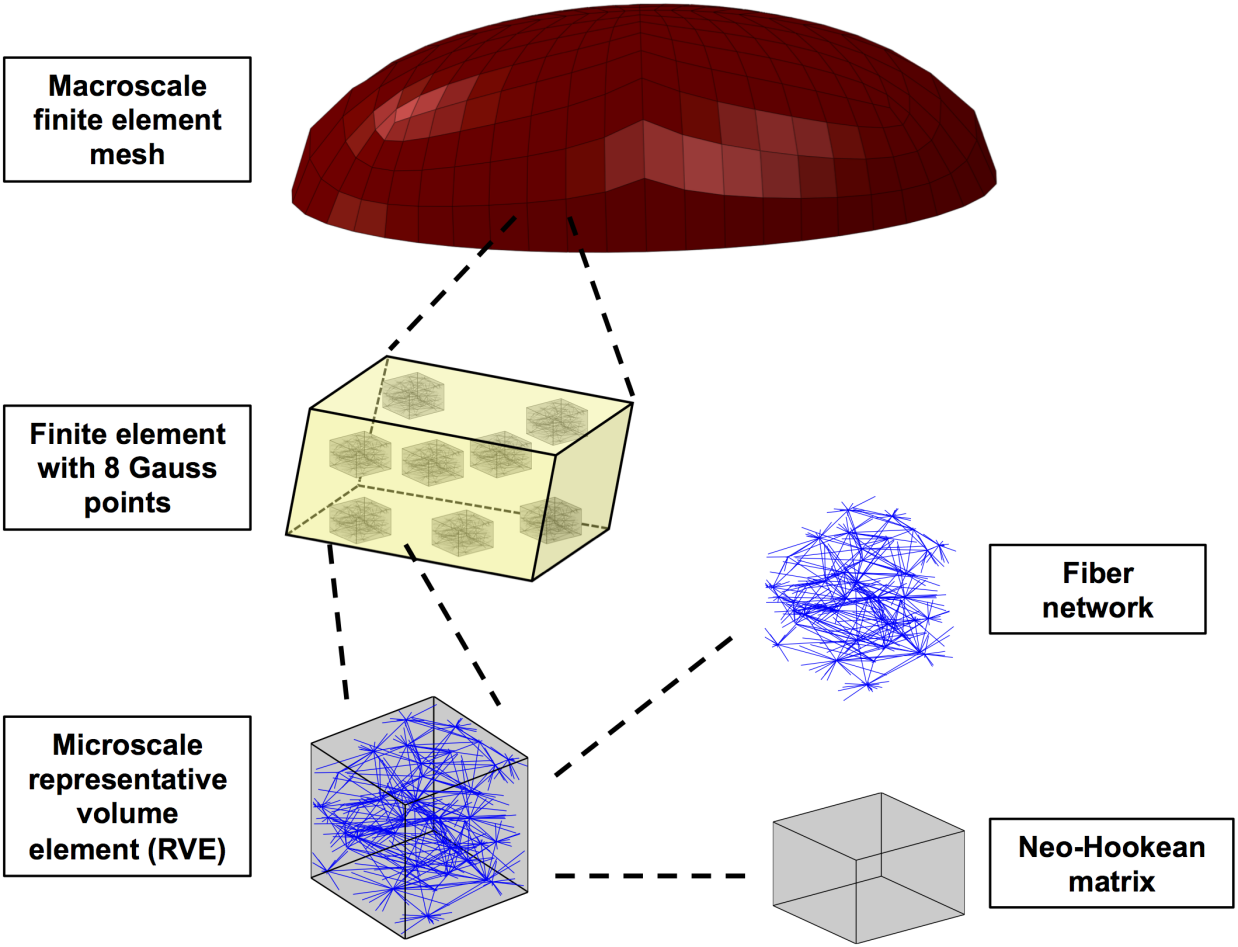
Multiscale model diagram. The multiscale model of the PC includes a macroscale finite-element mesh with representative volume elements (RVEs) of microscale fiber networks and neo-Hookean matrix.

A multiscale scheme [16–18] was used to model the response of the PC to indentation. The method is summarized here and described in detail elsewhere [16–18]. A finite-element model at the macroscopic level was coupled with representative volume elements (RVE), each comprised of a fiber network, at the microscopic scale (Fig 2). Each finite element contained eight Gauss points, each with an associated RVE. Each RVE contained a network of 500–700 fibers in a constrained mixture (cf. [19,20]) with a nearly incompressible neo-Hookean matrix. In this study, each element received a unique set of fiber networks depending upon its location within the mesh.

Macroscopic-level deformations were passed down to the microscopic level, the networks within each RVE were thus stretched, and the force exerted by each fiber, *F*, was calculated using the fiber constitutive equation

$$F=\frac{A}{B}(\text{exp}(BE_{f})-1)$$ (1)

where *A* is a measure of fiber stiffness, *B* is a measure of fiber nonlinearity, and *E*_*f*_ is the fiber Green strain computed from the fiber stretch, *λ*_*f*_,

$$E_{f}=0.5(\lambda_{f}^{2}-1)$$ (2)

From the fiber forces on the RVE boundaries, the volume-averaged Cauchy stress at each Gauss point within the macroscopic element was calculated as

$$S_{ij}^{macro}=\frac{1}{V}\int_{V}S_{ij}^{micro}\;dV=\sum_{bc}x_{i}F_{j}$$ (3)

where *V* is the RVE volume, $S_{ij}^{micro}$ is the microscale stress, *bc* refers to summation over all network nodes on the RVE boundary, *x*_*i*_ is the boundary fiber cross-link *i*-coordinate, and *F*_*j*_ is the force acting on the boundary fiber cross-link by the fiber in the *j*-direction. The averaged stress balance was given as [17]

$$S_{ij,i}=\frac{1}{V}\oint_{dV}(S_{ij}^{micro}-S_{ij}^{macro})u_{k,i}n_{k}dS$$ (4)

where *u*_*k*_ is the RVE boundary displacement and *n*_*k*_ is the normal vector to the RVE boundary. The displacements were updated until the stress balance Eq (4) had equilibrated. Simulations were run on 64 cores at the Minnesota Supercomputing Institute.

### Mesh Generation

All finite-element meshes were generated in ABAQUS. A mesh convergence study on the isolated PC problem gave average errors of 5% in nodal displacement between the coarsest mesh and the finest mesh (which was used in the study). Based on this result and our previous studies [16], we expect our numerical results to have errors of at most 5%.

### Microstructural Model Specifications

**Fig 3:**
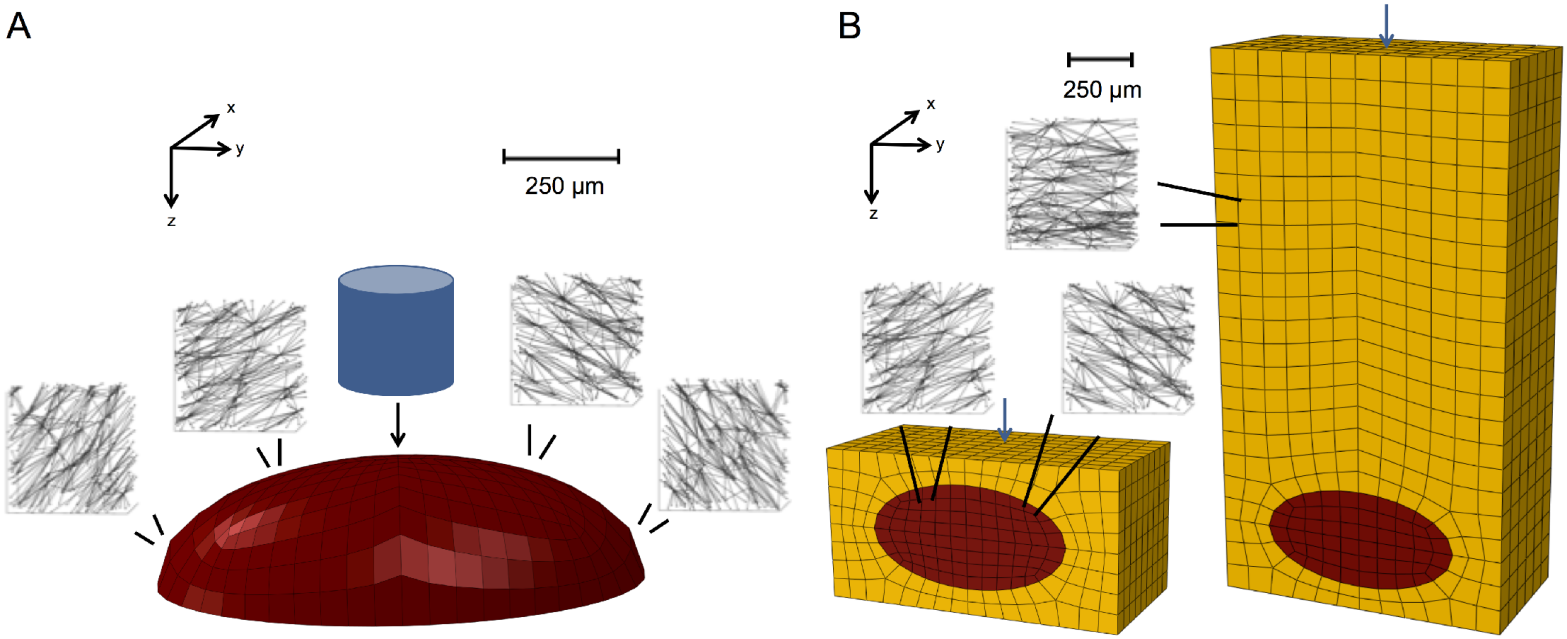
Finite-element meshes and representative networks. (A) The isolated PC model was populated with circumferentially-aligned Delaunay networks and indented with an indenter of diameter 250μm. (B) The epidermis and dermis PC models were populated with circumferentially-aligned Delaunay networks in the PC elements (red) and transversely isotropic Delaunay networks in the skin elements (gold). Single nodes on the surface of the meshes were indented.

Delaunay networks were created to populate the RVEs within the multiscale model. To capture the anisotropy of the collagenous lamellae within the PC, the networks for each finite element were aligned with the PC’s surface (Fig 3A). In the embedded models, the networks populating skin elements were made transverse orthotropic and aligned with the surface of the skin to reflect dermal collagen organization [21].

Material constants *A* and *B* for eq (2) were set at 114 μN and 10 respectively, obtained from fitting the multiscale model to data from a published study on dermal mechanics [22]. The Poisson’s ratio for the neo-Hookean matrix was set at ν = 0.47 for the simulations to model a nearly-incompressible matrix. The matrix shear modulus was set at G = 4.2 kPa [16]. The properties of the Pacinian corpuscle lamellae are not known, and estimates of the modulus of the lamellar layers have ranged from 1 KPa [23] to 0.5 MPa [11]. Neither of those bounds was based on a published mechanical measurement of the properties of collagen fibers within the PC; the small value was estimated based on assumed high compliance of the basement membrane, and is consistent with an anecdotal reference from the literature ([24] cited by [10], but no published data in [24]), and the latter was based on arterial wall. Recent work [23] has found that using a low modulus for the lamellar stiffness gives more accurate predictions of PC response in the high-frequency range, but collagen fibers have been found to have moduli in the MPa range [25], as have basement membranes from the renal tubule [26] and ocular lens [27–29]. It is thus clear that a better theoretical and structural description is needed, and it is likely that the choice of modulus will depend on the structure of that model. Since the emphasis of this work was on the effect of anisotropy, not on differences in stiffness between the corpuscle and the surrounding skin (which could be a very important factor and which should be explored in future studies), we chose to give the fibers in our PC model the same properties in as the fibers in the skin.

### Macroscale Model Specifications: Isolated Corpuscle

The isolated Pacinian corpuscle was meshed as a half-ellipsoid, with a major axis of length 1 mm and a minor axis of length 0.5 mm. The PC model contained 2984 hexagonal elements. The isolated PC model was subjected to 10 μm indentation by a 250 μm diameter indenter to simulate the experiments performed by Güçlü *et al*. and the associated finite-element model [7]. The indenter displaced nodes vertically, as shown in Fig 3A.

For consistency with the Güçlü experiments, the nodal displacements in the isolated PC model were analyzed. The displacement of nodes located along the top 100 μm of the z-axis was calculated after 10 μm indentation. Strain (*ε*_*yy*_) along the long axis of the PC was calculated for comparison to the embedded model.

### Macroscale Model Specifications: Embedded Corpuscle

#### Model preparation

A tissue-embedded PC was meshed as an ellipsoid with a major axis of 1 mm and a minor axis of 0.5 mm inside of a rectangular prism (Fig 3B). For the epidermis model, the domain was 1.5 mm x 1.5 mm x 0.75 mm, with the center of the PC located 0.375 mm beneath the surface. For the dermis model, the domain was 1.5 mm x 1.5 mm x 2.75 mm, with the center of the PC located 2.375 mm beneath the surface. The epidermis and dermis models had 2928 and 4380 hexahedral elements, respectively. The long axis of the PC was parallel to the surface of the skin.

The embedded PC models were subjected to 10 μm indentations at the surface of the skin. Nodes on the surface of the skin were indented individually in different simulations to construct the receptive field of the PC.

To observe the effect of PC orientation with respect to the skin surface, a dermally-embedded PC was modeled with its long axis perpendicular to the skin surface. This vertical PC model was subjected to 10 μm indentations at the skin surface.

#### Prediction of axial stretch on the PC

When the PC is mechanically stimulated, a receptor potential is produced and increases until a threshold is reached, and an action potential is initiated [4,5,10]. This study used the working model that stretch along the long axis of the receptor, and thus along the nerve fiber at the center of the PC, causes stretch-gated cation channels along the axon to open and initiates the response of the PC [30].

Since the deformations involved are very small, the response of the PC to indentation for the embedded models was determined by calculating the linear strain along the long axis (y-axis) of the PC to simulate stretching of the axon,

$$\varepsilon_{yy}=\frac{l_{y}-l_{yo}}{l_{yo}}$$ (5)

where *l*_*y*_ is the length of the PC along the y-axis after 10 μm indentation and *l*_*yo*_ is the initial length of the PC along the y-axis. Length was calculated as the distance between the y-coordinates of two nodes located at the interface of PC and skin elements on the y-axis of the PC. In the case of the vertically-aligned dermally-embedded PC, the linear strain along the long axis Eq (5) was measured with respect to the z-axis.

## Results

### Isolated Corpuscle

**Fig 4:**
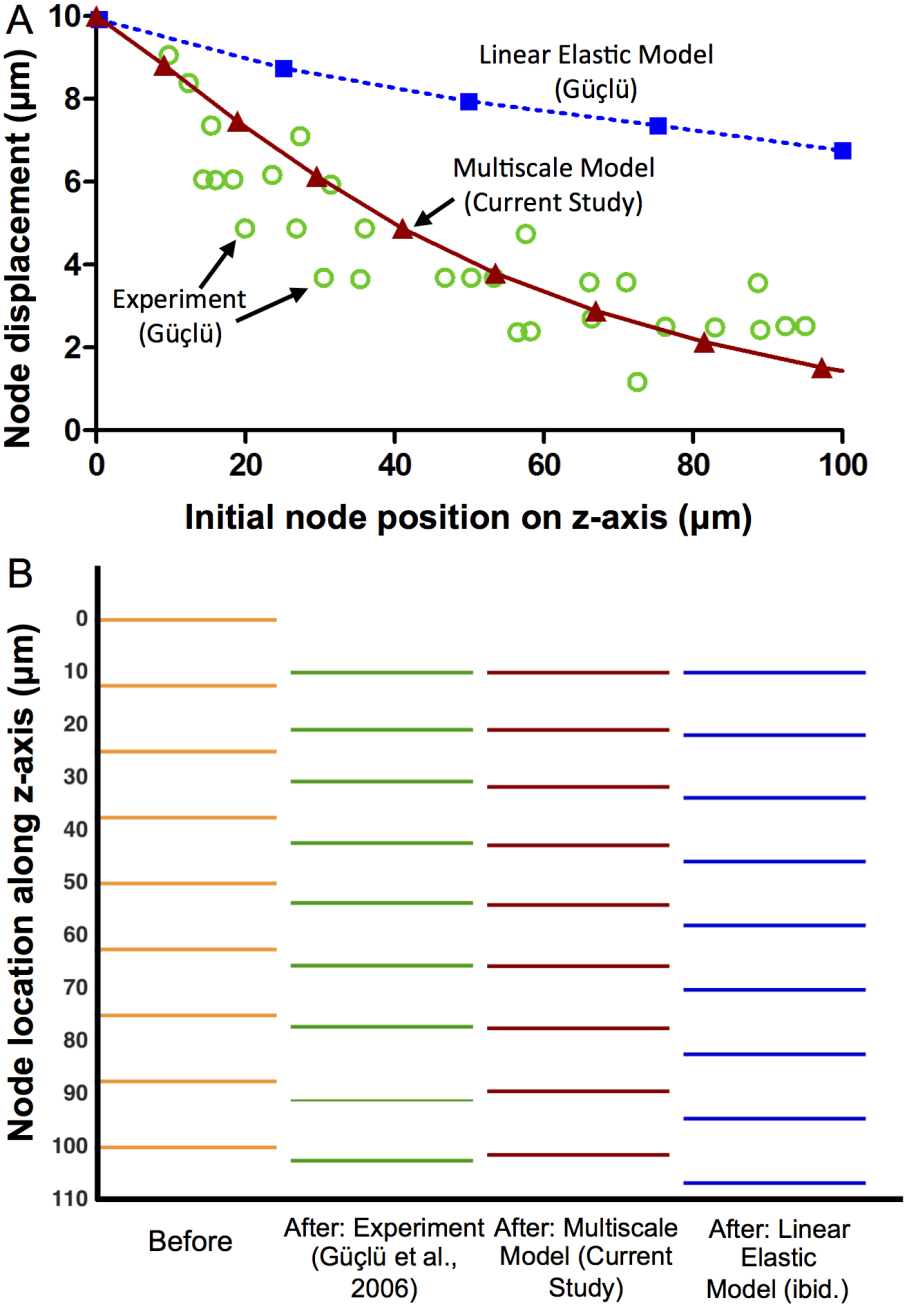
Comparison between model and experiments [7]. (A) Rapid, nonlinear drop in displacement with distance from the indenter site observed experimentally (green circles, from Güçlü) is matched by the current model (red triangles, solid line) but not by a linear elastic model (blue squares, dotted line; from Güçlü). (B) To visualize the nonlinearity, lines were drawn to represent the initial (first column) and final (second column) of the nodes in the indentation experiment. The multiscale model (third column) matched the experimental data much better than the linear elastic model (fourth column). These results confirm Güçlü’s observation that a linear elastic model cannot capture the response of the PC and demonstrate the ability of a structurally-motivated anisotropic model to do so.

The isolated PC was subjected to a 10 μm indentation with an indenter of diameter 250 μm to mimic the experiment and simulation of Güçlü *et al*. The multiscale model captured the nonlinear trend in displacement seen in the experimental data and not predicted by an isotropic linear elastic model (Fig 4A). The multiscale model populated with isotropic Delaunay networks rather than circumferentially-aligned networks also produced results similar to Güçlü *et al*.’s isotropic linear elastic model. Fig 4B shows the displacement of nodes along a cross section through the x-z plane of the PC at y = 0. As seen in Fig 4B, the multiscale model predicted a nonlinear spacing between nodes, with a greater nodal gap occurring with increasing depth.

**Fig 5:**
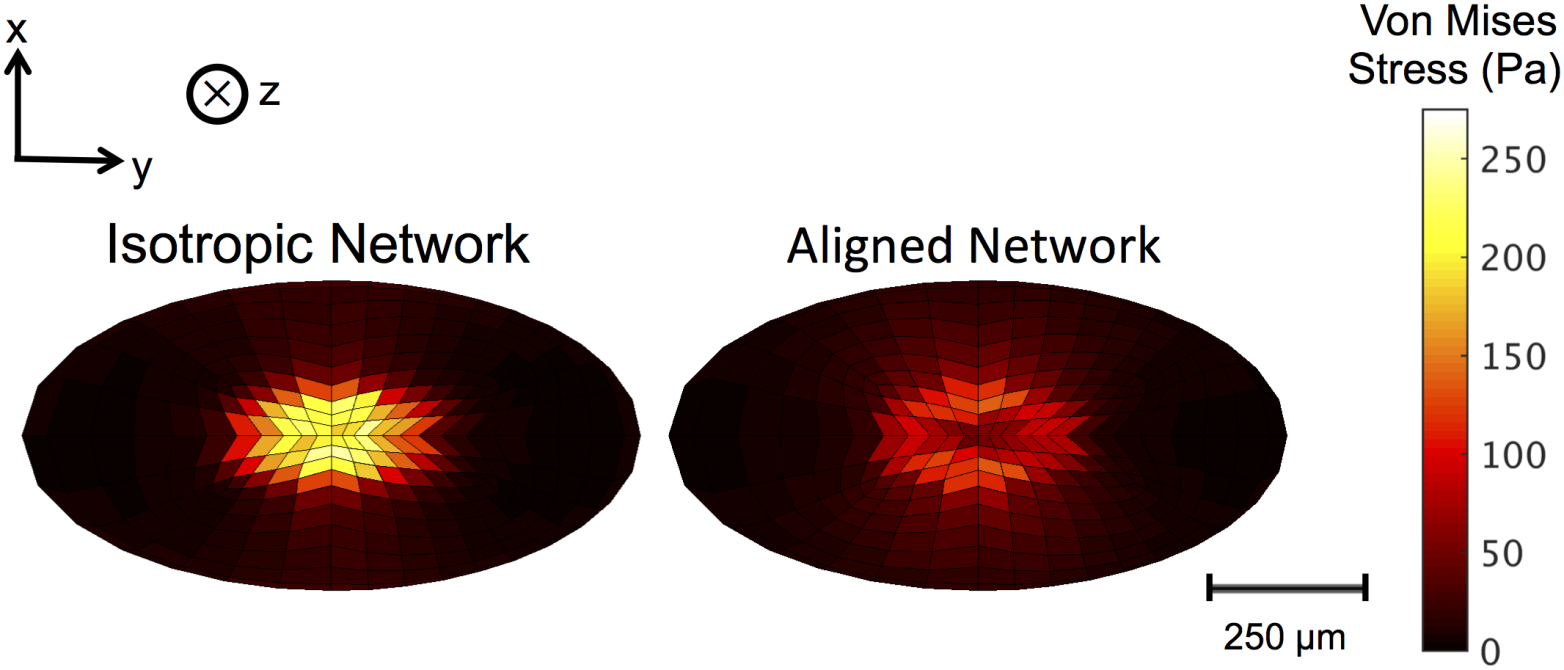
Von Mises stress in isotropic and aligned network cases. Von Mises stress along the top (surface layer) of the PC after 10 μm indentations (in the +z direction) with a 250μm diameter indenter for simulations run with isotropic networks (left) or circumferentially-aligned networks (right). The isotropic network case shows higher stress around the indenter than that shown in the aligned network case.

The Von Mises stress was calculated at each element in the PC after 10 μm indentation for the cases of isotropic networks and circumferentially-aligned networks. In Fig 5, the isotropic case shows stress of approximately 2x higher magnitude around the indenter than those shown for the circumferentially-aligned case.

**Fig 6:**
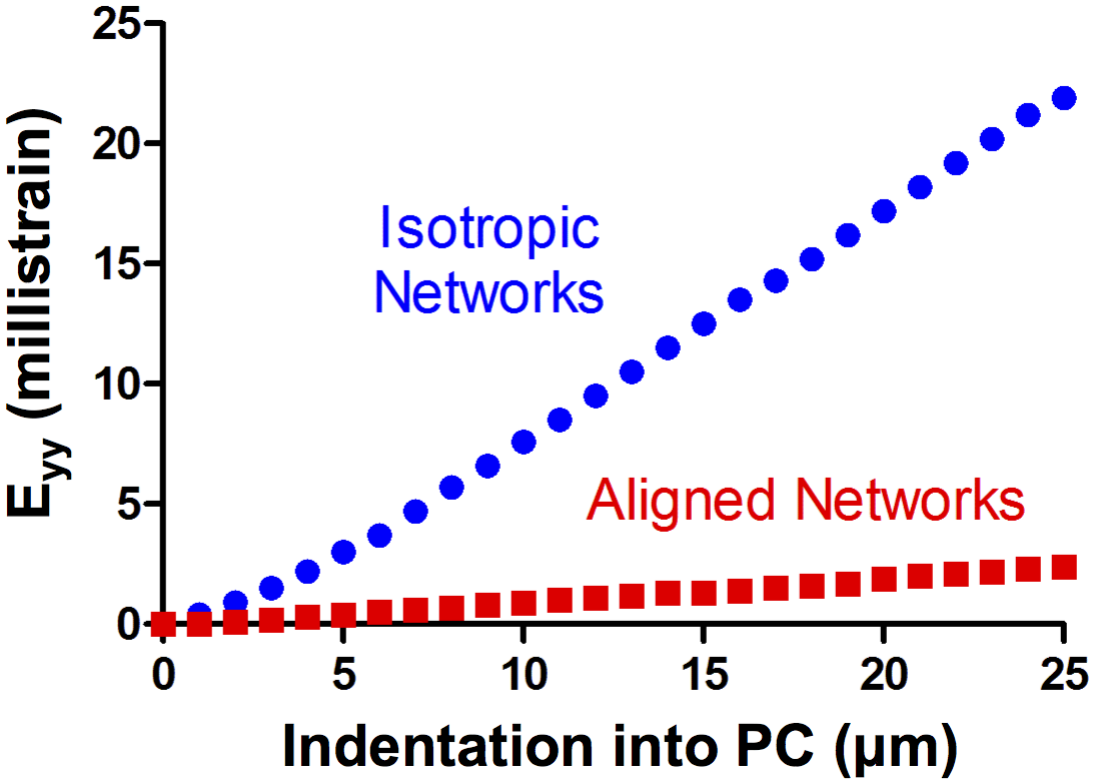
Long-axis strain in isotropic and aligned network cases. The strain along the long axis of the PC was calculated after 25 steps of 1 μm indentation. The cases of circumferentially-aligned (red squares) network and isotropic (blue circles) networks are shown. While the long-axis strain increases monotonically with indentation into the PC in both network cases, the isotropic network case showed higher strain than the aligned network case.

The strain along the long axis of the PC was calculated over 25 steps of 1 μm indentation. As seen in Fig 6, the strain along the long axis of the axon increased with indentation into the PC. The strain calculated for the isotropic network case was approximately 10x higher than that calculated for the circumferentially-aligned network case.

### Embedded Corpuscle

**Fig 7:**
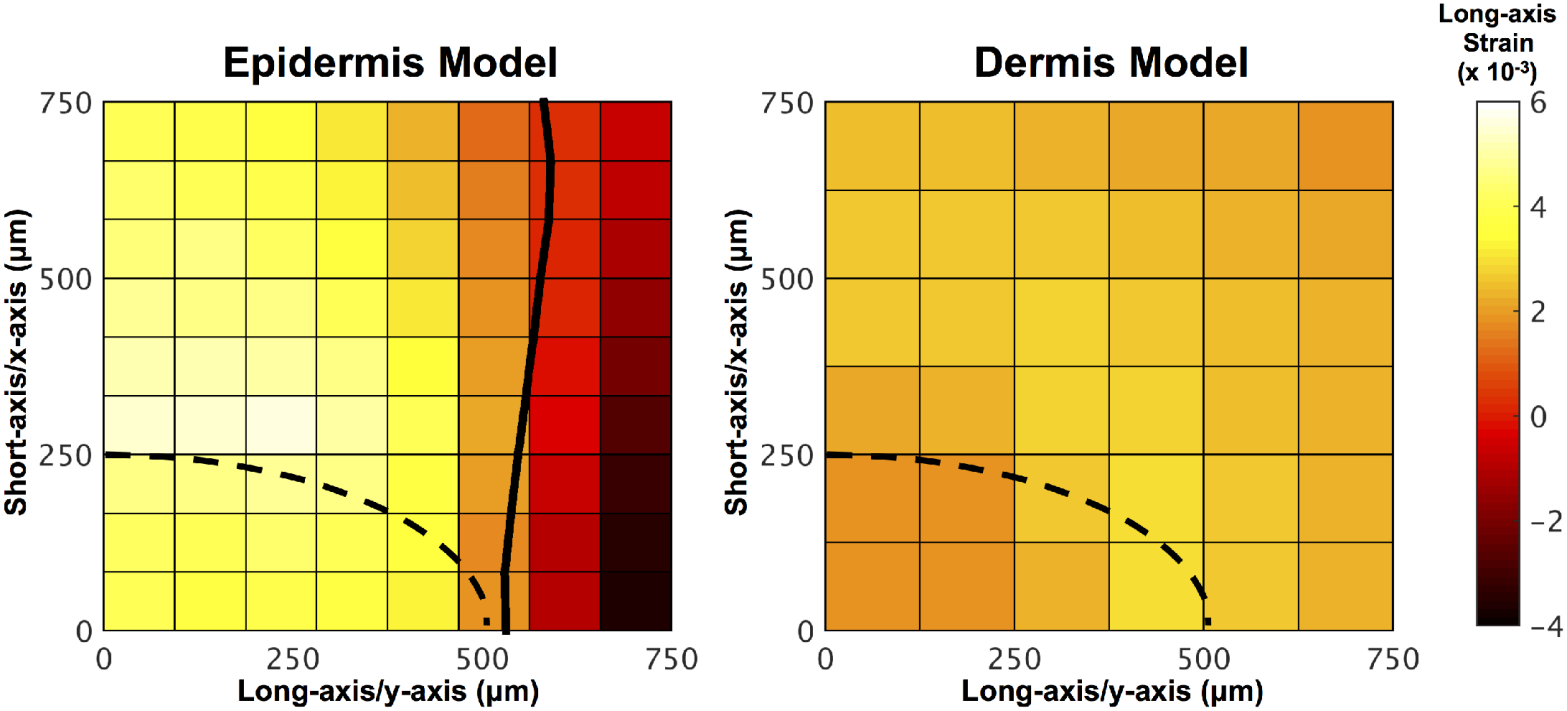
PC long-axis strain resulting from surface indentation at various nodes. Surface indentation sensitivity plot showing the long-axis strain along the PC resulting from 10 μm indentation at various nodes along the surface of the epidermis (left) and dermis (right) models. The dotted black line indicates the position of a quarter of the PC beneath the surface of the skin. The solid black line on the epidermis plot indicates the contour line for zero strain, which is the strain value below which the neurite would not be expected to respond to indentation.

The long-axis strain along the PC resulting from indentation at various nodes along the surface was also compared for the epidermis and dermis models (Fig 7). The epidermal PC model showed large strain in response to loading directly above the PC that dropped off quickly as the indenter moved away from the PC. This drop-off implies more spatial sensitivity and thus a smaller receptive field. The dermal PC model shows less indenter position dependence and thus a larger receptive field.

**Fig 8:**
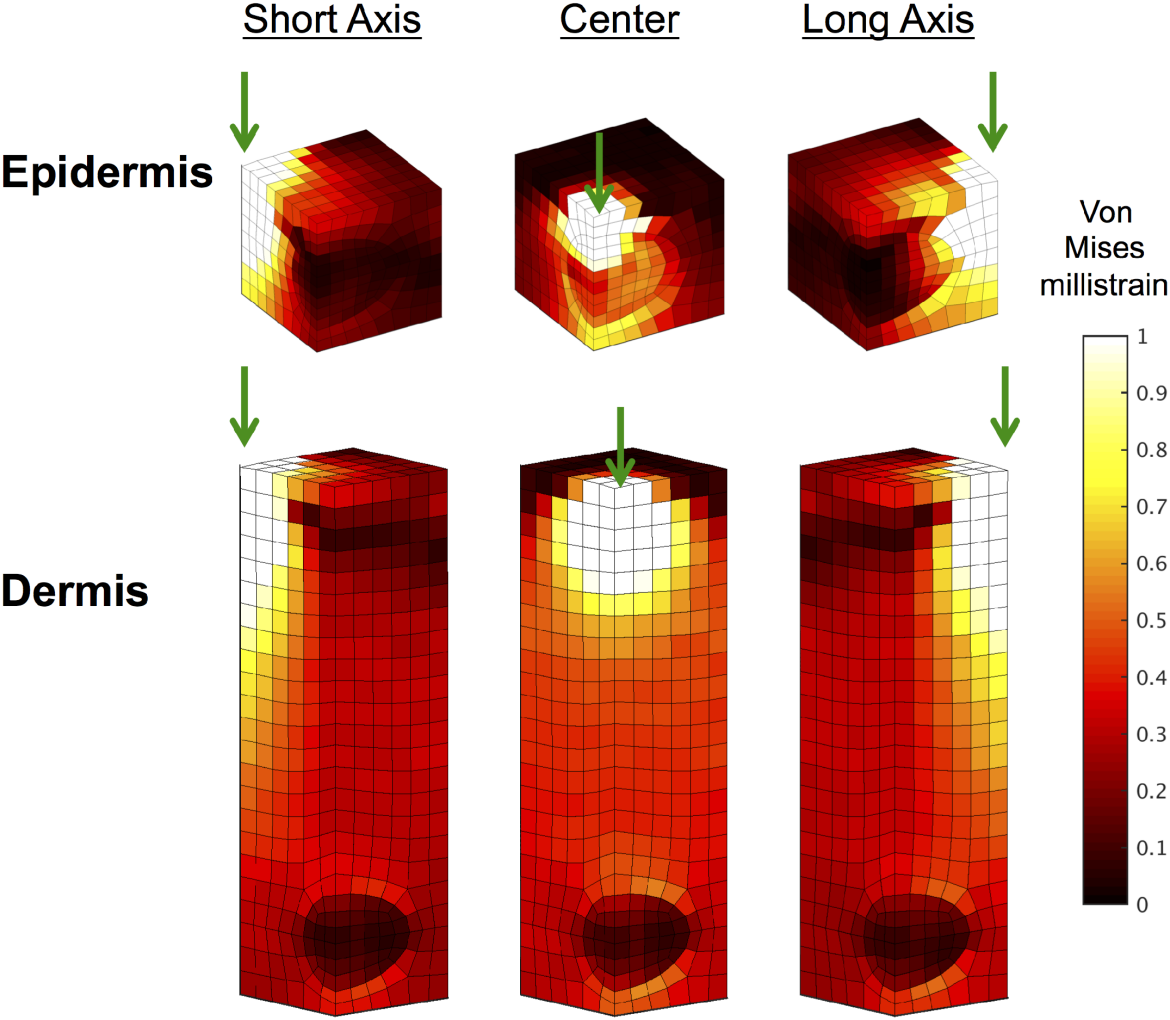
Von Mises strain resulting from surface indentation at different locations. The Von Mises strain (millistrain) at each element in the epidermis (top) and dermis (bottom) cases calculated after 10 μm indentation at three different locations on the surface (750 μm down the short axis of the PC, directly above the center of the PC, 750 μm down the long axis of the PC). The green arrows indicate the location of indentation. The large-strain region around the indenter reaches the epidermally-located PC but does not penetrate to the depth of the dermally-located PC. Only one quarter of the embedded PC mesh is shown due to symmetry.

The Von Mises strain was calculated within every element in the dermal- and epidermal-embedded cases after 10 μm indentation for indentations above the center of the PC and 750 μm down its long and short axes. As seen in Fig 8, the Von Mises strain in the PC in the dermis case shows little variation when the structure is indented at different locations. In both cases, PC strain is less than that of the immediately surrounding tissue because of its greater degree of anisotropy. The strain in the PC in the epidermis case shows greater variations with indenter location.

**Fig 9:**
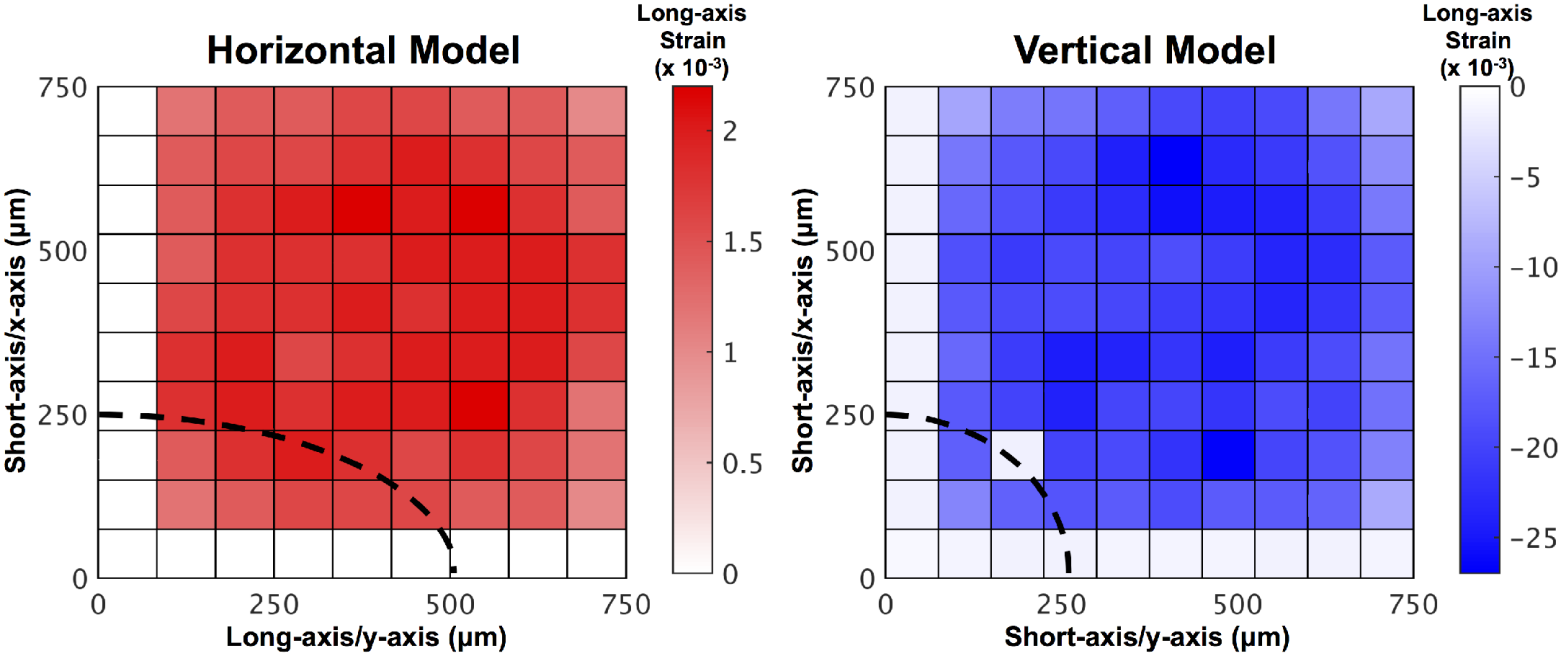
PC long-axis strain for horizontal and vertical PC alignment in skin mesh. Surface indentation sensitivity plot showing the long-axis strain along the PC resulting from 10 μm indentation at various nodes along the surface of the skin in a PC oriented with its long axis parallel to the surface of the skin (left, Horizontal) and its long axis perpendicular to the surface of the skin (right, Vertical). Both models contain a dermally-embedded PC. The dotted black line indicates the position of a quarter of the PC beneath the surface of the skin. The scale bars indicate the long-axis strain values for each model.

The long-axis strain along the PC resulting from 10 μm indentation at various nodes along the surface of the skin was compared for horizontally-aligned and vertically-aligned PCs embedded within a dermal mesh (Fig 9). The horizontal PC model showed positive strains or no strain resulting from indentation. The vertical PC model always showed negative strains in response to indentation. This result shows that indentation within the receptive field of a horizontally-aligned PC always results in positive axial stretch of the neurite. Indentation of the vertical model does not result in neurite stretch.

## Discussion

This study used a multiscale finite-element model to determine that the structure of the PC is an important contributor to the nonlinear behavior of the receptor. In addition, it showed that the deep dermal location of the PC provides it with lower spatial sensitivity.

Several factors must be considered when interpreting our results. First, the mechanical stimulus was a fixed indentation into the PC with no transient effects. As such, this study addresses the location and magnitude of the stimulus but it does not take stimulus frequency into account when determining the PC mechanical response, as others have [11]. A static model was chosen as suitable for comparison with experiment [7], but it would not be appropriate for simulating the vibrotactile response. While Hubbard [31] also investigated PC mechanics, the results from the current paper cannot be directly compared to that study, which placed the PC within a hinged apparatus rather than stimulating with a vertical indenter. Therefore, the experiments performed by Güçlü *et al*. were used to validate the current model. Second, the PC was treated as incompressible, and no fluid movement was allowed within the PC even though such flow is known to be important [11]; thus, our model must be interpreted as the instantaneous response of the PC. The time-dependent response of the PC is necessary to address, as it is crucial to the PC’s role as a high-pass filter to vibration [5,11], so insights from the current study should center on instantaneous response. Also, the experimental PC literature [4,5,32] focuses appropriately on the use of directly applied sinusoidal displacements to elicit the response of the PC to vibratory stimuli, providing a rich data set on the dynamical response of the PC. A model of PC mechanics should include a dynamical component (fluid flow, viscoelasticity, or both) to account for the phase difference that can occur between skin and PC stimulation, and also between PC stimulation and receptor response.

The mechanical model used in this study also simplified the structure of the PC to account only for anisotropy within the receptor and not for its specific components and detailed structure. The receptor capsule is composed of concentrically-arranged collagenous lamellae through which mechanical forces are transduced. The lamellae consist of condensed cell layers separated by layers of pressurized fluid [33]. The structure of the capsule is believed to play an important role in determining which mechanical forces are transmitted to the axon [34]. Each lamella contains a single layer of flat squamous epithelial type cells, with interlamellar spacing increasing with distance from the inner core [10]. Tight junctions between cells within each lamella prevent fluid flow across lamellae, but flow within the fluid layer between adjacent lamellae is possible and is significant at low frequencies. Loewenstein and Skalak first proposed that the role of the PC capsule’s lamellar structure is that of a series of mechanical high-pass filters to shield the nerve fiber at the center of the receptor from low frequency, high amplitude stimuli [11]. To place this model in the broader context, it is more advanced than that of Güçlü, which is isotropic and linear elastic, but does not provide the single-lamellar-level description of Lowenstein and Skalak or of subsequent variations thereon [23,35]. The multi-scale approach of the current model, in which RVE’s are introduced with position-dependent fiber orientations, could be extended to more complex microstructures as greater structural information becomes available. It is also notable that the lamella-based models [11,23,35] can account for the apparent viscoelasticity of the tissue by incorporating interlamellar flow; our model would not be able to do so but could incorporate a continuous viscous contribution similar to that derived previously [33] in a homogenized model of the PC. Clearly, there is need for a more detailed microstructural model that can address other aspects of PC behavior and perhaps can resolve the disconnect between the high stiffness typical of collagenous tissues and the low stiffness reported experimentally [24] and used to describe vibrotactile mechanics of the PC [23].

**Fig 10:**
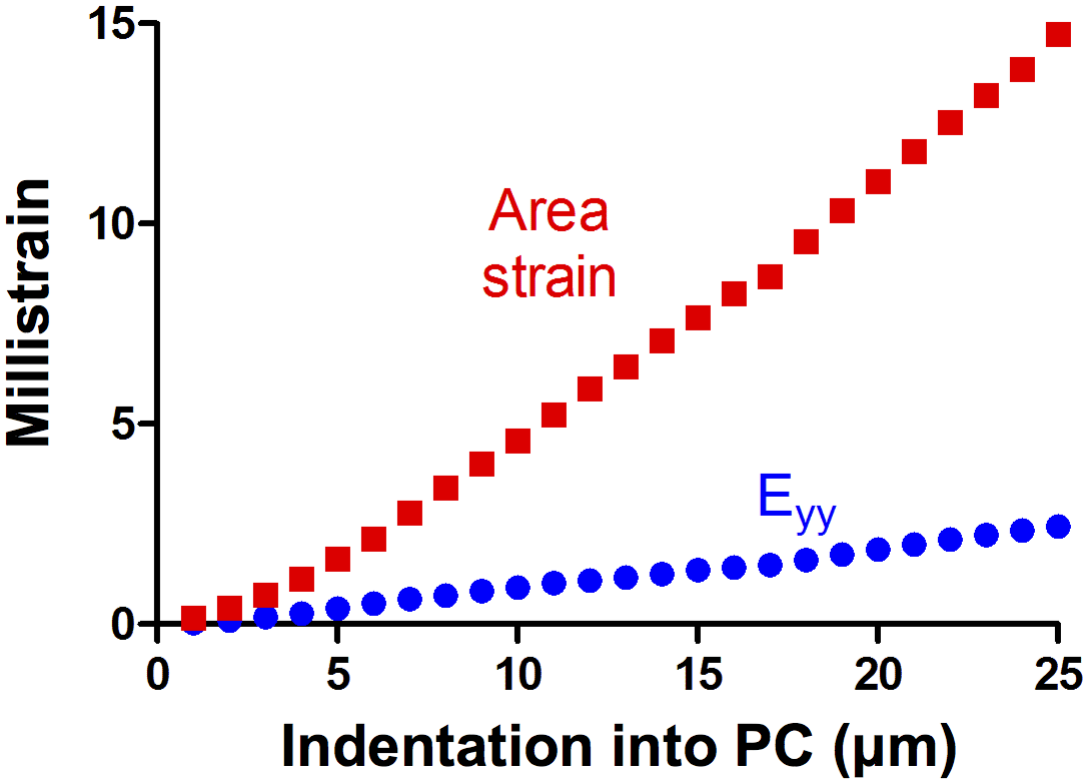
Comparison between long-axis strain and area strain. The long-axis strain of the PC (blue circles) and the area strain of the PC (red squares) were calculated for 25 steps of 1 μm indentation. Both long-axis strain and area change increase monotonically as the PC is indented.

The mechanical model presented in this study simplified the neurophysiology of the PC action potential generation into axial stretch of its central nerve fiber. The solid black line in Fig 7 indicates the division between the positive and negative stretch. Because the exact mechanism of axon excitation is unknown, it is possible that the neurite could also be stimulated during compression along the long axis. Long-axis compression could, for example, be experienced as positive stretch in other directions due to neurite incompressibility. Thus, while it is possible that long-axis compression could lead to axon excitation, only long-axis stretch was considered in this study. It was initially proposed by Gray & Ritchie [30] that sensory receptors respond to mechanical stimulation resulting from nerve stretch. After PC compression studies performed by Hubbard in 1958 were unable to measure a change in axon length within the error of measurement, other possible mechanical mechanisms for transduction were proposed [31]. Specifically, Hubbard proposed that a change in the ratio between the major and minor axes of the cross section of the nerve fiber leads to a change in surface area and thus membrane stretch. Fig 10 shows a comparison between the model of axon stretch along the long axis used in this study and the area change proposed by Hubbard. Both long-axis strain and area strain increase monotonically with indentation into the PC. The area change of the PC is approximately six times the long-axis strain after 25 μm of indentation.

The orientation of the PC with respect to the surface of the skin was analyzed in this study. The results presented in Fig 9 show that indentation within the receptive field of a horizontally-aligned PC always results in either axial stretch of the neurite or no neurite stretch. Indentation within the receptive field of a vertically-aligned PC always results in neurite compression. The assumption used in this study that PC action potential generation is the result of axial stretch of the neurite and not axial compression means that the vertically-aligned PC was never activated by static compression. This study only shows the results of two PC orientations to static compression. A vibratory stimulus would involve both application and removal of an indenter due to oscillations, creating a more complex field that would likely include long-axis stretch in both cases. Thus, it is expected that different results on PC orientation would be obtained from models of vibration. It has previously been shown [36] that the electrophysiological response of an intact PC or its decapsulated nerve terminal changes polarity as it is rotated 90 degrees along its long axis. The same study also showed that a decapsulated terminal reversed the polarity of its neural response when compressed horizontally to the nerve or vertically to the nerve and proposed that the bilateral arrangement of cells around the terminal is responsible for changing how compression is transmitted to the neurite in these different orientations. It has also been shown [37] that compression of an intact PC along its long axis requires a much stronger stimulus to cause depolarization than that required in compression along the short axis. The current study models the transmission of mechanical stimuli through the lamellae to the neurite, and showed differences with PC orientation, but does not address the electrophysiological effects reported by others [36,37]. A combined model (cf. [33,38]) could lend greater insight into the interaction between mechanical and electrophysiological events.

Incorporation of isotropic Delaunay networks into the PC multiscale model rather than circumferentially-aligned networks resulted in a lack of shape dependence similar to that observed in Güçlü’s finite-element study [7]. The current study thus confirms the finding that the ellipsoidal shape of the PC is not per se responsible for the observed mechanical behavior. In addition, the use of isotropic Delaunay networks further confirmed Güçlü‘s result that a homogeneous isotropic model of the PC cannot predict the experimentally observed displacement pattern in response to indentation. This study showed that the internal anisotropic structure is an important factor leading to the nonlinear displacements through the PC. The nonlinear reduction in displacement of lamellae located closer to the central core is in agreement with the hypothesis that the lamellar structure can help protect the nerve from large deformations under large skin surface loads.

The current model bases the mechanical properties of the networks representing the skin on data from uniaxial mechanical tests on dermis [22]. There are many factors that can influence the mechanics of skin, which can vary with anatomical location, proximity to blood vessels, thickness, body weight, hair-cycle stage, skin disease, and experimental conditions such as humidity [39–42]. Specifically, mechanical and structural properties such as viscoelasticity and anisotropy of skin can vary with age and anatomical location [42]. The mechanical properties of a skin sample are influenced by structural components such as the collageneous fiber network and the presence of different layers, which exhibit different mechanical properties, within the skin [35,40,41,43]. Selecting different data for fitting of the skin element mechanical properties used in this study would likely change the quantitative results of the current study due to differences in the aforementioned factors. The overall qualitative results of this study, however, are not expected to change. The mechanics of skin can also vary depending on the type of load applied, as skin behaves differently under compression and tension [39,40]. In vivo skin can also be in different amounts of tension depending on anatomical location, the body position, and the individual [42]. All of the aforementioned factors could be considered in future models, with the current model functioning as a basis for subsequent studies and as a low-order model of skin behavior.

Past experiments have shown that the isolated PC is a highly sensitive mechanoreceptor with nanometer sensitivity [2]. The simulations performed in this study model the sensitivity of an isolated and embedded PC to micrometer-scale indentations. Sensitivity thresholds of the PC have been reported previously as 3 nm applied directly to the PC and 10 nm applied to the surface of the skin [2]. In the current study, the smallest indentation tested on the isolated PC was 1 μm, which corresponds to an amplitude of approximately 7 μm applied to the skin after comparison of the strain along the long axis of the PC in the isolated and dermis-embedded models. This ratio could change for a more anatomically detailed model, in which multiple receptors rather than just one are located within the dermis. There are currently no published experimental data on the mechanics on a PC embedded in skin.

While this work focused on the PC, its results can also provide insight into other cutaneous mechanoreceptors. The embedded PC simulations showed that a PC located in the dermis of the skin was able to replicate the low spatial sensitivity of a PC *in vivo*. The PC embedded in the epidermis had a higher spatial sensitivity within the receptive field tested in the simulations. Receptors located closer to the surface of the skin, such as the Meissner corpuscle and Merkel cell-neurite complex, show decreased receptive fields and thus increased spatial sensitivity to mechanical events on the skin surface [44]. The current study could also be expanded to include the different geometries and cutaneous locations of other mechanoreceptors.
